# Thymic Stromal Lymphopoietin Induces Migration in Human Airway Smooth Muscle Cells

**DOI:** 10.1038/srep02301

**Published:** 2013-07-29

**Authors:** Naresh Singh Redhu, Lianyu Shan, Hesam Movassagh, Abdelilah S. Gounni

**Affiliations:** 1 Department of Immunology, Faculty of Medicine, University of Manitoba, Winnipeg, MB, Canada; 2 These authors contributed equally to this work.; 3 Current address: Department of GI/Nutrition, Boston Children's Hospital, Harvard Medical School, Boston, MA, 02115, USA.

## Abstract

Airway remodeling due to increased airway smooth muscle (ASM) mass, likely due to enhanced migration and proliferation, has been shown to be highly associated with decline in lung function in asthma. Thymic stromal lymphopoietin (TSLP) is an IL-7-like, pro-allergic cytokine that has been shown to be necessary and sufficient for the development of allergic asthma. Human ASM (HASM) cells express TSLP receptor (TSLPR), the activation of which leads to enhanced release of proinflammatory mediators such as IL-6, CCL11/eotaxin-1, and CXCL8/IL-8. We show here that TSLP induces HASM cell migration through STAT3 activation since lentiviral-shRNA inhibition of STAT3 abrogated the TSLP-induced cell migration. Moreover, TSLP induced multiple cytoskeleton changes in HASM cells such as actin polymerization, cell polarization, and activation of small GTPase Rac1. Collectively, our data suggest a pro-migratory function of TSLP in ASM remodeling and provides better rationale for targeting TSLP/TSLPR pathway for therapeutic approaches in allergic asthma.

Airway remodeling is a cardinal feature in allergic asthma that culminates in the form of abnormal epithelium, subepithelial thickening, mucus gland hypertrophy, alteration in extracellular matrix deposition, and increase in airway smooth muscle (ASM) mass. ASM remodeling has been shown to be one of the most crucial factors that correlate with decreased lung function in asthma^1^. Proposed mechanisms of ASM remodeling include enhanced migration, proliferation (hyperplasia), and increased size (hypertrophy) of ASM cells. Although the source of migrating ASM cells in airways remain a matter of debate (e.g. from circulating fibrocytes, the original deeper ASM layer, or from bone marrow/lung-derived mesenchymal stem cells), the process of migration has been proposed extensively to underlie, at least in part, the airway remodeling and thus the pathogenesis of asthma^2^. Although these structural changes are known to cause substantial airflow limitation in asthma^3^, they are not reversed by currently availably asthma therapies. Thus, there is a stern need to identify the novel triggers and mechanisms of ASM migration.

Thymic stromal lymphopoietin (TSLP) is a pro-allergic, IL-7-like hematopoietic cytokine that has been shown to be necessary for the development of allergic asthma in some animal models^4^. TSLP exerts its biologic effects through a heterodimeric TSLP receptor (TSLPR) composed of TSLPR subunit and IL-7Rα^5^. Animal models overexpressing TSLP exhibit enhanced susceptibility to and the intensity of allergic airway disease while the mice lacking TSLP receptor (TSLPR−/−) had considerably attenuated disease, discussed in^6^. The identification of triggers of TSLP expression and its cellular targets is an emerging area of investigation. In airways, TSLP is produced by epithelial cells, mast cells, ASM cells, and fibroblasts^7^. TSLP activates immature CD11c+ dendritic cells (DC) to express the costimulatory ligand OX40L, which renders DCs to become mature and migrate to the draining lymph nodes. These DCs then stimulate the naive CD4+ T cells via binding to the OX40, to differentiate into inflammatory type 2 (Th2) cells producing IL-4, IL-5, IL-13, tumor necrosis factor (TNF), and no or little IL-10^8^. In addition, TSLP favors Th2 environment by directly enhancing the cell proliferation, STAT5 phosphorylation, and expression of anti-apoptotic factor Bcl-2 in Th2 cells^9^. TSLP is highly expressed in ASM bundles from asthma^10^ and COPD patients^11^. HASM cells were recently recognized to express a functional heterodimeric receptor TSLPR^12^; and the activation of HASM via TSLPR leads to proinflammatory cytokine IL-6, chemokines CXCL8/IL-8 and CCL11/eotaxin-1 release, and increase in intracellular Ca^2+^ 7,12,13. Interestingly, TSLP was shown to induce migration in DCs^14^, suggesting a novel physiological function of TSLP that could potentially promote inflammation. However, the effect of TSLP on ASM migration remains unknown. We show here, for the first time, an increased HASM cell migration in response to TSLP stimulation, and uncover some of the putative signaling mechanisms involved in this process.

## Results

### TSLP induces migration in HASM cells

**Figure 1:**
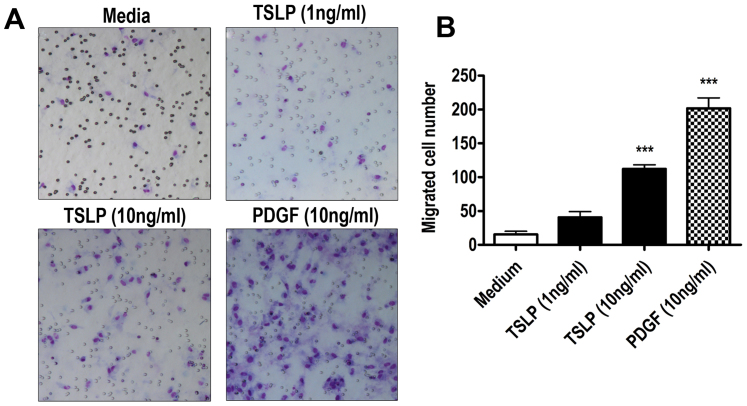
TSLP induces Human airway smooth muscle (HASM) cell migration. (A) HASM cells seeded in Boyden upper chamber were stimulated with TSLP (1, 10 ng/ml) and PDGF (10 ng/ml) in lower chamber, and migrated cells were analyzed by light microscope (magnification, x200). Migrated cells were quantitated and presented in (B) ***p < 0.001 vs medium control (*One-way ANOVA followed by Newman-Keuls Multiple Comparison Test*).

To assess whether TSLP can affect HASM cell migration, Boyden chamber assay for cell migration was performed on primary HASM cells. We found that the recombinant human TSLP (1–10 ng/ml) induces HASM cell migration (p < 0.001, n = 4, Fig. 1A). One of the physiological stimuli of HASM cell migration, platelet derived growth factor-BB (PDGF-BB) was used as a positive control which induced highly significant increase in the number of migrated HASM cells (p < 0.001, n = 4, Fig. 1A, B). Taken together, our data shows that TSLP can elicit HASM cell migration.

### TSLP-induced HASM cell migration requires STAT3 activity

**Figure 2:**
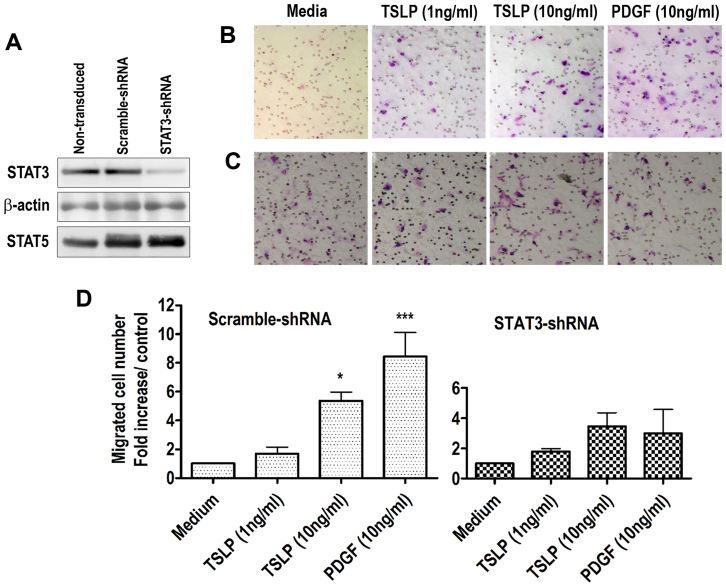
STAT3 mediates TSLP-induced HASM cell migration. (A) Lentiviral-shRNA transduction silenced the STAT3 expression in HASM cells as shown by Western blotting. Scramble- (B) and STAT3-shRNA (C) silenced HASM cells were analyzed for migration. (D) TSLP-induced HASM cell migration was quantified and presented as fold increase relative to unstimulated control. *p < 0.05, ***p < 0.001, *One-way ANOVA followed by Newman-Keuls Multiple Comparison Test*.

We then sought to investigate the underlying signaling mechanisms of TSLP-induced cell migration. We have previously established that Signal transducer and activator of transcription 3 (STAT3) but not STAT5 is phosphorylated and only STAT3 activation is required for TSLP-induced HASM cell synthetic functions^12^. Therefore, we performed lentiviral-shRNA-mediated inhibition of STAT3 in our HASM cells^12^ and (See *Methods* section). As shown in Fig. 2A lentiviral-shRNA inhibited the total STAT3 expression significantly. We observed that the TSLP-induced cell migration was nearly abolished in STAT3-shRNA-transduced cells (p > 0.05 compared to unstimulated control, n = 3, Fig. 2C, D). The scramble-shRNA-transduced HASM cells exhibited normal migration response (p < 0.05, n = 3, Fig. 2B, D) which was similar to wild-type/nontransduced HASM cells in response to TSLP stimulation in Fig. 1, suggesting the specificity of STAT3 targeting by shRNA transduction. It has been shown earlier that STAT3 serves as a signature element in PDGF signaling, at least in HASM cells. In agreement with these observations^15^, STAT3 inhibition also led to complete loss of PDGF-induced HASM cell migration in our study (Fig. 2C, D), also strengthening our experimental approach. Therefore, our data clearly shows that TSLP and PDGF induce HASM cell migration in a STAT3-mediated manner.

### TSLP induces cytoskeletal changes to induce HASM cell migration

**Figure 3:**
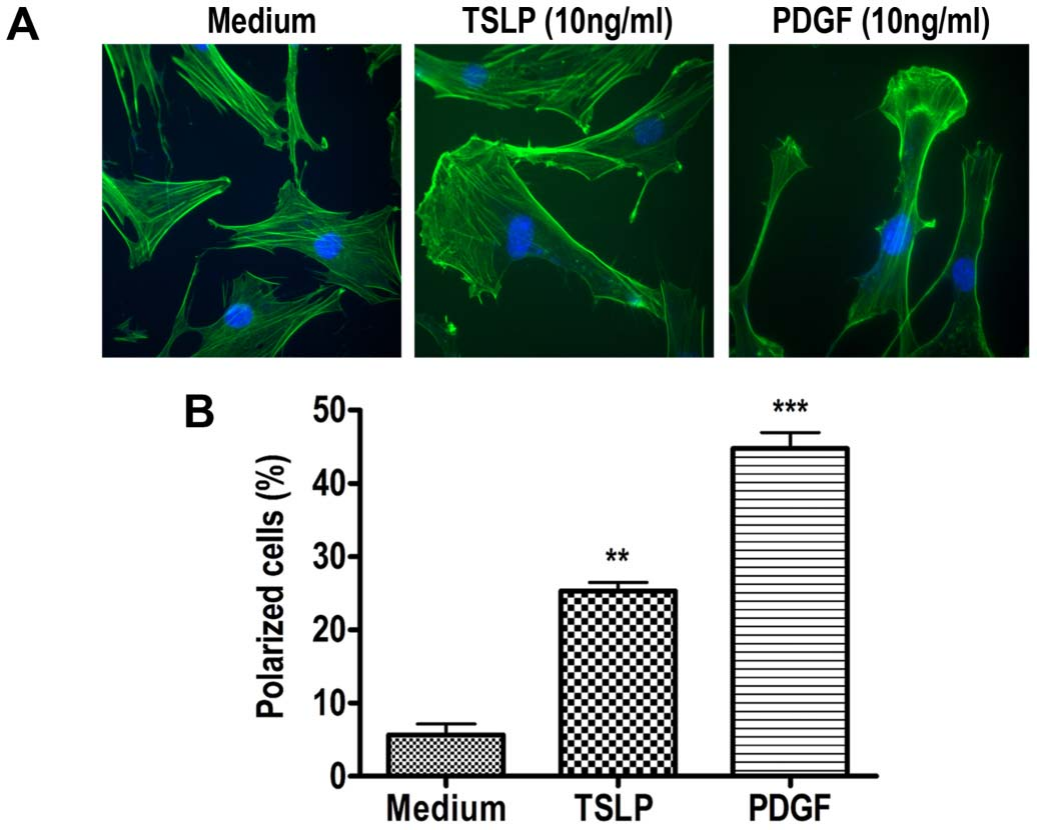
TSLP induces cytoskeleton changes in HASM. (A) Polymerized actin in TSLP or PDGF-stimulated HASM cells was analyzed by phalloidin staining, quantified as percent polarized cells. **p < 0.01, ***p < 0.001 vs unstimulated control; *One-way ANOVA and Newman-Keuls Multiple Comparison Test*.

Reorganization of actin cytoskeleton is a fundamental mechanism in cell motility and migration. To further unravel the potential mechanisms of actin substructures involved in migration process, we employed cytoskeleton staining of HASM cells by using phalloidin-Alexa Fluor® 488 (Invitrogen, Burlington, ON) (See *Methods* section). As shown in representative micrographs of phalloidin-stained cells in Fig. 3A; whereas untreated cells showed flattened shapes, both TSLP (10 ng/ml), and PDGF (10 ng/ml)-treated cells showed polarized shapes. In other words, TSLP and PDGF induced lamellipodia structure formation, indicative of polymerized actin required for cell migration^16^. Consistent with the proportion of migrated cells in Fig. 1 and 2, TSLP induced polarization in 25.29 ± 1.180% and PDGF in 44.75 ± 2.195% of the cells (p < 0.0.01 and p < 0.001, respectively; n = 3) compared with that in unstimulated 5.670 ± 1.470% cells (Fig. 3 B).

### TSLP induces Rac1 activity in HASM cells

**Figure 4:**
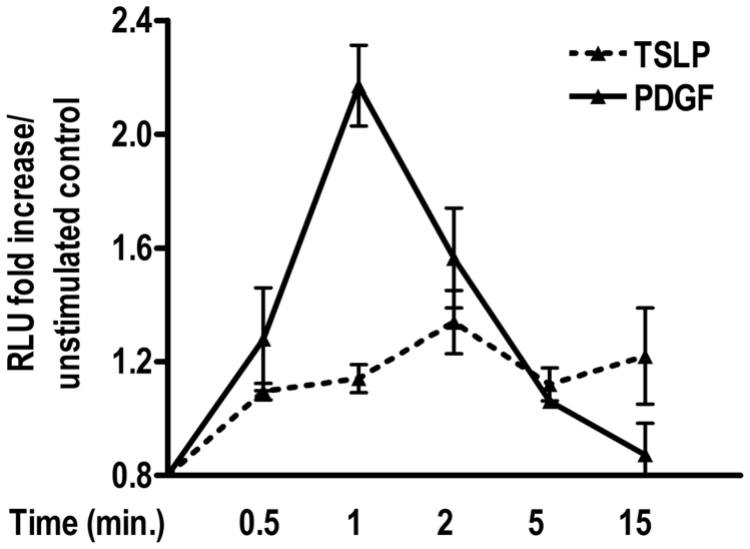
TSLP and PDGF induce Rac1 activation in HASM cells. GTPase Rac1 activity in TSLP-stimulated HASM cells was measured by G-LISA Rac1 activation assay kit. The Rac1 activity is presented as relative luminescence units (RLU) over unstimulated control.

The primary requirement for lamellipodia formation is the activation of small guanosine triphosphate hydrolase (GTPase) Rac1 within minutes of extracellular stimuli^16^. In order to confirm the role of Rac1 in microscopic changes observed in actin cytoskeleton, we performed luminescence-based G-LISA™ assay (Cytoskeleton Inc., Denver, CO) in the cell lysates prepared upon TSLP stimulation (10 ng/ml) (See *Methods* section). As shown in Fig. 4, TSLP stimulation induced marked increase in Rac1 activity over the unstimulated control. As a positive control, PDGF also led to enhanced Rac1 activity in HASM cells (Fig. 4). Collectively, our data suggest that TSLP-induced HASM cell migration may occur potentially via Rac1 activation and actin cytoskeleton rearrangement.

## Discussion

The data presented here uniquely shows a pro-migratory function of TSLP in HASM cells. TSLP-induced HASM cell migration was dependent upon STAT3 activity as shown by lentiviral-shRNA-mediated STAT3 inhibition experiments. Further analysis showed that TSLP invokes necessary cytoskeleton rearrangements such as formation of lamellipodia, cell polarization, and enhanced activity of small GTPase Rac1. Collectively, we show that TSLP may induce pro-migratory function in human ASM cells.

Migration of smooth muscle occurs during tube formation of hollow organs including blood vessels, gastrointestinal tract, and airways^17^. A similar phenomenon of migration of smooth muscle of airways has been proposed to happen in response to tissue injury, inflammation, and underlies airway remodeling^17^. Although ASM cells from asthmatic subjects have been shown to exhibit increased proliferative capacity than from non-asthmatics^18^, it is currently unknown whether the migratory potential is also altered between two groups. However, stimulation of ASM with some growth factors and cytokines, found to be increased in asthmatic airways such as IL-8/CXCL8, TGF-β, PDGF-BB, and extracellular matrix components collagens, fibronectin, and laminin, promotes cell migration^19^. By showing that TSLP can induce HASM cell migration, we add a new pro-migratory factor to this list. Although our data does not essentially confirm a role for TSLP in the pathogenesis of airway remodeling, we provide at least one mechanism through which HASM cell migration could occur. Interestingly, we also found that TSLP can induce a modest level of proliferation in HASM cells (*N. S. Redhu, L. Shan, and A. S. Gounni, unpublished observations*). Therefore, this data provides a strong rationale for further studies in animal models to assess the role of TSLP in airway smooth muscle remodeling. In support of our proposal, a recent study using house dust mite (HDM) allergen-induced asthma model showed that neutralization of TSLP with anti-TSLP mAb reversed the airway inflammation, prevented structural alterations, and decreased the pro-remodeling cytokine TGF-β expression and AHR to methacholine^20^. However, further studies are required to confirm a specific role of TSLP in increased ASM mass in context of cell migration while considering other important phenomenon of ASM hypertrophy and hyperplasia.

Although TSLP has recently emerged as a direct player in initiation of allergic inflammatory responses, it was initially recognized as a growth factor and mitogen for pro-B cell line Ba/F3^21^. It should also be noted that TSLP expression is increased in COPD and allergic asthma airways including the smooth muscle tissue^10^,^11^,^22^. It is now clear that TSLP can stimulate myeloid and lymphoid cells, eliciting the proliferation of naïve T cells, pro- and pre-B cells, and migration of dendritic cells (DCs)^23^,^14^,^24^. TSLP also rescued the eosinophils from apoptosis and enhanced the surface adhesion molecules, inflammatory gene expression, and cell chemotaxis^25^. We have earlier demonstrated a critical role of TSLP in wound repair, proliferation, and migration of airway epithelial cells in allergic asthma^26^. TSLP-induced migration of HASM cells in this study extends the role of TSLP as a potent pro-remodeling regimen in airway disease. Although not fully understood, signaling mechanisms activated by TSLP are becoming increasingly known. STAT3 is one of the critical signaling molecules that have been implicated in promoting allergic inflammation. In particular, a predominant role of STAT3 was shown in studies where epithelial STAT3 disruption led to controlled airway eosinophilia^27^, and IL-17A induced the eotaxin-1/CCL11 (an eosinophil mobilizing chemokine) expression in HASM cells^28^. Although both STAT3 and STAT5 have been shown to be activated in response to TSLPR activation, STAT5 is considered as a signature signaling dock for TSLP in hematopoietic cells^6^. However, STAT3 but not STAT5 is phosphorylated and only STAT3 activation was required for TSLP-induced HASM cell synthetic functions^12^. In current report, TSLP-induced migration was abrogated in STAT3-silenced HASM cells, suggesting that STAT3 serves as an essential signaling mediator.

The process of cell migration is initiated by activation of receptors such as G protein-coupled receptors (GPCRs), receptor tyrosine kinases (RTK), and integrins which trigger the remodeling of cytoskeleton, discussed in context of HASM cell migration extensively in^17^. Actin polymerization is a proximal event that propels the leading edge of the cell towards the stimulus. The small G proteins such as Ras, Rac, Rho, and Cdc42 are prominent early signaling elements that promote cell migration. In our results, TSLP clearly induced polarized state of HASM cells, and formation of lamellipodia confirmed the initial events required for cell migration. Furthermore, TSLP induced a significant level of Rac1 activity in HASM cells. Although Rac family members are known to be involved in proliferation of ASM cells^29^, their role in ASM cell migration is unknown^17^. Moreover, Rac1 and Rac2 have been shown earlier to mediate PDGF-induced migration in vascular smooth muscle (VSM) cells^30^. Our data shows that PDGF-induced Rac1 activation may indeed be required for HASM cell migration. In summary, this data demonstrates that TSLP and PDGF-BB may induce HASM cell migration by inducing key structural changes including actin polymerization and Rac1 activity.

Taken together, as the appreciation for the role of TSLP in initiation and/or perpetuation of airway inflammation is growing, current report underscores a potential pro-remodeling function of TSLP. TSLP-induced HASM cell migration involves STAT3 activity, actin polymerization, cell polarization, and Rac1 activity. It is highly likely that locally produced TSLP in airways (from ASM, epithelium, and/or mast cells), besides activating the DCs to shape the inflammatory Th2 differentiation, can act in an autocrine/paracrine manner to directly induce local ASM and epithelial tissue remodeling, prominent in allergic asthma. Therefore, strategies targeting the TSLP, its receptor, or signaling components may additionally be considered for trials in airway remodeling.

## Methods

### Reagents

Recombinant human TSLP and PDGF-BB were purchased from R&D Systems (Minneapolis, MN). FBS was from HyClone Laboratories (Logan, UT). DMEM, Ham's F12, trypsin-EDTA, antibiotics (penicillin, streptomycin), were from Invitrogen Life Technologies (Grand Island, NY). All other reagents were procured from Sigma-Aldrich Canada Ltd. (Oakville, ON), unless specified.

### Preparation and stimulation of human airway smooth muscle (HASM) cells

Three different sources of HASM cells were used. Written informed consent was obtained from the tissue donors, and this study was approved by the research ethics committee of the University of Manitoba, Winnipeg, Canada. Both hTERT-immortalized and primary human bronchial smooth muscle (HBSM) cells were prepared as described previously^12^,^31^,^32^. Primary human tracheal smooth muscle (HTSM) cells were kindly provided by Dr. Thomas Murphy, Department of Pediatrics, Duke University Medical Center, Durham, NC. HTSM cells were cultured and maintained in a similar method as primary HBSM cells^32^,^33^. In all the experiments, primary HASM cells were used at passages 2–6, and hTERT cells at passages 10–30. Sub-confluent HASM cells were growth arrested and synchronized by serum deprivation for 48 h in Ham's F-12 medium containing 1X ITS (5 μg/ml human recombinant insulin, 5 μg/ml human transferrin, 5 ng/ml selenium) (Invitrogen), and antibiotics (100 U/ml penicillin and 100 μg/ml streptomycin). Cells were then stimulated in fresh FBS-free F-12 medium containing recombinant human TSLP (1, 10 ng/ml), PDGF-BB (10 ng/ml), or medium alone for indicated time periods.

### Boyden chamber cell migration assay

HASM cell migration was analyzed by using Boyden chamber assay as described previously^34^. Briefly, 48 h serum-deprived cells were detached from the culture plate using trypsin-EDTA solution (Invitrogen Canada Inc., Burlington, ON) and resuspended in Ham's F12 medium containing antibiotics, and 1X ITS. A polycarbonate membrane of 8 μm pore size (Neuroprobe, Gaithersburg, MD, USA) was coated with 0.01% collagen type-I in 0.01N HCl solution (Sigma). A 50 μl aliquot of HASM cells (5 × 10^4^ cells/ml) was added to the upper chamber of modified Boyden chamber apparatus (Neuroprobe). In the lower chamber, TSLP (1, 10 ng/ml) or PDGF-BB (10 ng/ml) were added as chemoattractant to the same media as the upper chamber. After 4 h of incubation at 37°C in humidified 5% CO_2_ incubator, the membranes were peeled-off. Cells on the upper side of the membrane were scraped off and the cells migrated to the lower side were fixed and stained with Hemacolor® stain set (EMD Millipore, Billerica, MA, USA). The number of migrated cells was counted in four-five random fields under X20 magnification by phase contrast microscope (Carl Zeiss Canada Ltd., Toronto, ON).

### Lentivirus-mediated STAT3-shRNA transduction in HASM cells

Lentiviral transduction of STAT3-short hairpin (sh) RNA (clone ID: V2LHS_262105) in HASM cells was performed as described earlier^12^. The average transduction efficiency was greater than 95%, analyzed by flow cytometry using the turbo green fluorescent protein (tGFP) as the marker for cell sorting (data not shown) and^12^. STAT3 expression in lentivirus-transduced cells was significantly reduced as shown by Western blotting (Figure 2A). Mock and lentiviral-STAT3-shRNA transduced HASM cells were cultured in the presence of TSLP (1, 10 ng/ml), PDGF-BB (10 ng/ml), or medium alone. Cell migration was assessed by Boyden chamber assay as described above.

### Phalloidin staining to measure actin polymerization

Immunofluorescence and confocal laser scanning microscopy was performed as we described previously^12^. To minimize the activation of cytoskeleton by nonspecific stimuli, physical manipulation of slides was kept to minimum. In brief, HASM cells were grown on 24-well glass slides (Nalge Nunc International, Naperville, IL) up to semi-confluence and stimulated with TSLP (10 ng/ml), PDGF (10 ng/ml) or media alone for 5–30 min. Slides were then fixed with 4% paraformaldehyde for 30 min, and stained with phalloidin- Alexa Fluor 488 (Invitrogen) for 30 min at room temperature in dark and analyzed by confocal microscopy.

### Measurement of GTPase Rac1 activity

Levels of small GTPase Rac1 were measured by a luminescence based G-LISA Rac1 activation assay biochem kit (Cat # BK126; Cytoskeleton, Denver, CO, USA). Briefly, 48 h serum-deprived HASM cells were cultured in presence of TSLP (10 ng/ml) and PDGF (10 ng/ml) and cell lysates were prepared at the indicated times by following the G-LISA kit manufacturer protocol. Luminescence in cell lysates was measured and relative luminescence units (RLU) were determined from experiments performed at least in duplicate.

### Statistical analysis

All the data were obtained from experiments performed at least in triplicate. Statistical analysis was performed by using GraphPad Prism Software Version 3.02 for Windows (GraphPad Software, San Diego, CA, USA). Data between groups was compared by using one-way analysis of variance (ANOVA) followed by Newman-Keuls multiple comparison test. P values < 0.05 were considered statistically significant.

## Author Contributions

N.S.R. wrote the main manuscript text, N.S.R. and L.S. performed the data analysis, L.S. and A.S.G. designed the experiments, L.S. and H.M. performed the experiments, and A.S.G. provided the reagents, conceived the study design, and critically reviewed, provided feedback and approved the final version of manuscript.

## References

[b1] Kaminska M. *et al.* Airway remodeling in subjects with severe asthma with or without chronic persistent airflow obstruction. J Allergy Clin Immunol 124, 45–51 e41–44 (2009). 19481790 10.1016/j.jaci.2009.03.049

[b2] Bentley J. K. & Hershenson M. B. Airway smooth muscle growth in asthma: proliferation, hypertrophy, and migration. Proc Am Thorac Soc 5, 89–96 (2008). 18094090 10.1513/pats.200705-063VS PMC2645305

[b3] Pepe C. *et al.* Differences in airway remodeling between subjects with severe and moderate asthma. J Allergy Clin Immunol 116, 544–549 (2005). 16159622 10.1016/j.jaci.2005.06.011

[b4] Ziegler S. F. The role of thymic stromal lymphopoietin (TSLP) in allergic disorders. Curr Opin Immunol 22, 795–799 (2010). 21109412 10.1016/j.coi.2010.10.020 PMC3032269

[b5] Park L. S. *et al.* Cloning of the murine thymic stromal lymphopoietin (TSLP) receptor: Formation of a functional heteromeric complex requires interleukin 7 receptor. J Exp Med 192, 659–670 (2000). 10974032 10.1084/jem.192.5.659 PMC2193276

[b6] Roan F. *et al.* The multiple facets of thymic stromal lymphopoietin (TSLP) during allergic inflammation and beyond. J Leukoc Biol 91, 877–886 (2012). 22442496 10.1189/jlb.1211622 PMC3360473

[b7] Redhu N. S. & Gounni A. S. Function and mechanisms of TSLP/TSLPR complex in asthma and COPD. Clin Exp Allergy 42, 994–1005 (2012). 22168549 10.1111/j.1365-2222.2011.03919.x

[b8] Ito T. *et al.* TSLP-activated dendritic cells induce an inflammatory T helper type 2 cell response through OX40 ligand. J Exp Med 202, 1213–1223 (2005). 16275760 10.1084/jem.20051135 PMC2213234

[b9] Kitajima M., Lee H. C., Nakayama T. & Ziegler S. F. TSLP enhances the function of helper type 2 cells. Eur J Immunol 41, 1862–1871 (2011). 21484783 10.1002/eji.201041195 PMC3124605

[b10] Kaur D. *et al.* Mast cell-airway smooth muscle crosstalk: the role of thymic stromal lymphopoietin. Chest 142, 76–85 (2012). 22052771 10.1378/chest.11-1782 PMC3418864

[b11] Zhang K. *et al.* Constitutive and inducible thymic stromal lymphopoietin expression in human airway smooth muscle cells: role in chronic obstructive pulmonary disease. Am J Physiol Lung Cell Mol Physiol 293, L375–382 (2007). 17513456 10.1152/ajplung.00045.2007

[b12] Shan L. *et al.* Thymic stromal lymphopoietin receptor-mediated IL-6 and CC/CXC chemokines expression in human airway smooth muscle cells: role of MAPKs (ERK1/2, p38, and JNK) and STAT3 pathways. J Immunol 184, 7134–7143 (2010). 20483734 10.4049/jimmunol.0902515

[b13] Smelter D. F. *et al.* Thymic stromal lymphopoietin in cigarette smoke-exposed human airway smooth muscle. J Immunol 185, 3035–3040 (2010). 20660708 10.4049/jimmunol.1000252 PMC3681514

[b14] Fernandez M. I. *et al.* The human cytokine TSLP triggers a cell-autonomous dendritic cell migration in confined environments. Blood 118, 3862–3869 (2011). 21772055 10.1182/blood-2010-12-323089

[b15] Simeone-Penney M. C. *et al.* PDGF-induced human airway smooth muscle cell proliferation requires STAT3 and the small GTPase Rac1. Am J Physiol Lung Cell Mol Physiol 294, L698–704 (2008). 18310224 10.1152/ajplung.00529.2007

[b16] Disanza A. *et al.* Actin polymerization machinery: the finish line of signaling networks, the starting point of cellular movement. Cell Mol Life Sci 62, 955–970 (2005). 15868099 10.1007/s00018-004-4472-6 PMC11924564

[b17] Gerthoffer W. T. Migration of airway smooth muscle cells. Proc Am Thorac Soc 5, 97–105 (2008). 18094091 10.1513/pats.200704-051VS PMC2645306

[b18] Johnson P. R. *et al.* Airway smooth muscle cell proliferation is increased in asthma. Am J Respir Crit Care Med 164, 474–477 (2001). 11500353 10.1164/ajrccm.164.3.2010109

[b19] Camoretti-Mercado B. Targeting the airway smooth muscle for asthma treatment. Transl Res 154, 165–174 (2009). 19766960 10.1016/j.trsl.2009.06.008 PMC2764304

[b20] Chen Z. G. *et al.* Neutralization of TSLP Inhibits Airway Remodeling in a Murine Model of Allergic Asthma Induced by Chronic Exposure to House Dust Mite. PLoS One 8, e51268 (2013). 23300949 10.1371/journal.pone.0051268 PMC3534685

[b21] Reche P. A. *et al.* Human thymic stromal lymphopoietin preferentially stimulates myeloid cells. J Immunol 167, 336–343 (2001). 11418668 10.4049/jimmunol.167.1.336

[b22] Ying S. *et al.* Expression and cellular provenance of thymic stromal lymphopoietin and chemokines in patients with severe asthma and chronic obstructive pulmonary disease. J Immunol 181, 2790–2798 (2008). 18684970 10.4049/jimmunol.181.4.2790

[b23] Scheeren F. A. *et al.* Thymic stromal lymphopoietin induces early human B-cell proliferation and differentiation. Eur J Immunol 40, 955–965 (2010). 20127673 10.1002/eji.200939419

[b24] Rochman Y. & Leonard W. J. Thymic stromal lymphopoietin: a new cytokine in asthma. Curr Opin Pharmacol 8, 249–254 (2008). 18450510 10.1016/j.coph.2008.03.002 PMC2518061

[b25] Wong C. K., Hu S., Cheung P. F. & Lam C. W. Thymic stromal lymphopoietin induces chemotactic and prosurvival effects in eosinophils: implications in allergic inflammation. Am J Respir Cell Mol Biol 43, 305–315 (2010). 19843704 10.1165/rcmb.2009-0168OC

[b26] Semlali A., Jacques E., Koussih L., Gounni A. S. & Chakir J. Thymic stromal lymphopoietin-induced human asthmatic airway epithelial cell proliferation through an IL-13-dependent pathway. J Allergy Clin Immunol 125, 844–850 (2010). 20236697 10.1016/j.jaci.2010.01.044

[b27] Simeone-Penney M. C. *et al.* Airway epithelial STAT3 is required for allergic inflammation in a murine model of asthma. J Immunol 178, 6191–6199 (2007). 17475846 10.4049/jimmunol.178.10.6191

[b28] Saleh A., Shan L., Halayko A. J., Kung S. & Gounni A. S. Critical role for STAT3 in IL-17A-mediated CCL11 expression in human airway smooth muscle cells. J Immunol 182, 3357–3365 (2009). 19265112 10.4049/jimmunol.0801882

[b29] Page K. *et al.* Regulation of cyclin D(1) expression and DNA synthesis by phosphatidylinositol 3-kinase in airway smooth muscle cells. Am J Respir Cell Mol Biol 23, 436–443 (2000). 11017907 10.1165/ajrcmb.23.4.3953

[b30] Doanes A. M., Irani K., Goldschmidt-Clermont P. J. & Finkel T. A requirement for rac1 in the PDGF-stimulated migration of fibroblasts and vascular smooth cells. Biochem Mol Biol Int 45, 279–287 (1998). 9678249 10.1080/15216549800202652

[b31] Redhu N. S., Saleh A., Halayko A. J., Ali A. S. & Gounni A. S. Essential role of NF-kappaB and AP-1 transcription factors in TNF-alpha-induced TSLP expression in human airway smooth muscle cells. Am J Physiol Lung Cell Mol Physiol 300, L479–485 (2011). 21148792 10.1152/ajplung.00301.2009

[b32] Redhu N. S. *et al.* IgE induces transcriptional regulation of thymic stromal lymphopoietin in human airway smooth muscle cells. J Allergy Clin Immunol 128, 892–896 e892 (2011). 21835441 10.1016/j.jaci.2011.06.045

[b33] Gosens R. *et al.* Role of caveolin-1 in p42/p44 MAP kinase activation and proliferation of human airway smooth muscle. Am J Physiol Lung Cell Mol Physiol 291, L523–534 (2006). 16617096 10.1152/ajplung.00013.2006

[b34] Zhang J. *et al.* Pentraxin 3 (PTX3) expression in allergic asthmatic airways: role in airway smooth muscle migration and chemokine production. PLoS One 7, e34965 (2012). 22529962 10.1371/journal.pone.0034965 PMC3329534

